# Supplementary material for: Fate of charge order in overdoped La-based cuprates
Source: NPJ Quantum Mater. 2023 Jan 19;8(1):7. doi: 10.1038/s41535-023-00539-w (PMC11041719; doi:10.1038/s41535-023-00539-w)
Supplement: Supplementary file 1 — Supplemental Material [file 41535_2023_539_MOESM1_ESM.pdf]

Supplementary Information

Fate of charge order in overdoped La-based cuprates

K. von Arx *et al.*

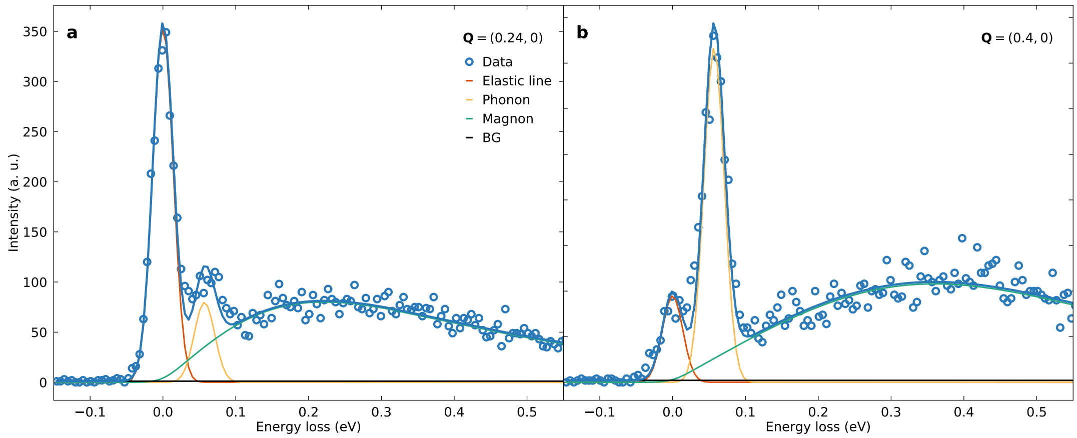

Supplementary Figure 1. **Raw RIXS spectra of LSCO $\mathbf{x = 0.145}$ and associated fits**. Representative raw RIXS spectra at **a** the charge order wave vector and **b** away from charge order recorded at 37 K. Solid lines indicate fits of respective elastic, phonon, magnon and background (BG) components of the spectra.

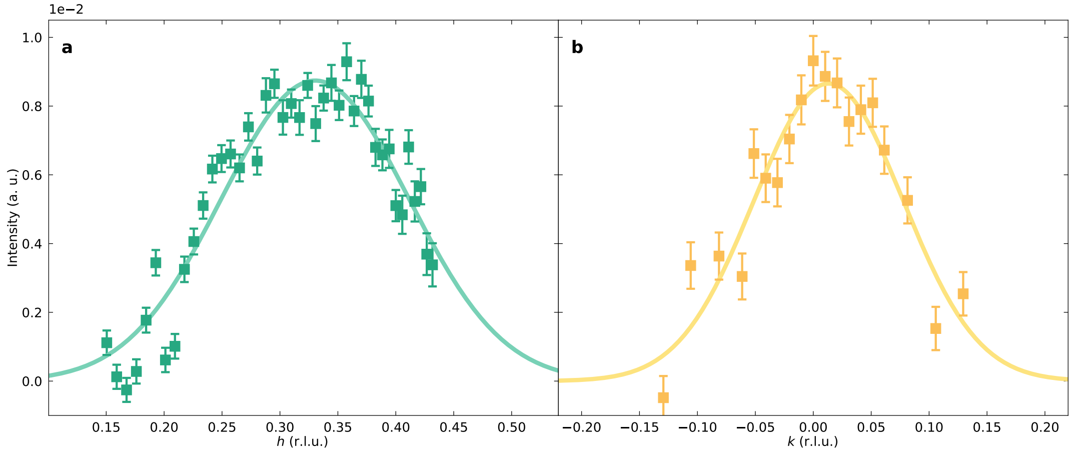

Supplementary Figure 2. **Elastic scattering recorded on LESCO $\mathbf{x = 0.21}$**. Longitudinal **a** and transverse **b** elastic scans through the charge order wave vector. Data was recorded with medium resolution at base temperature. Solid lines are Gaussian fits. Error bars are set by counting statistics.

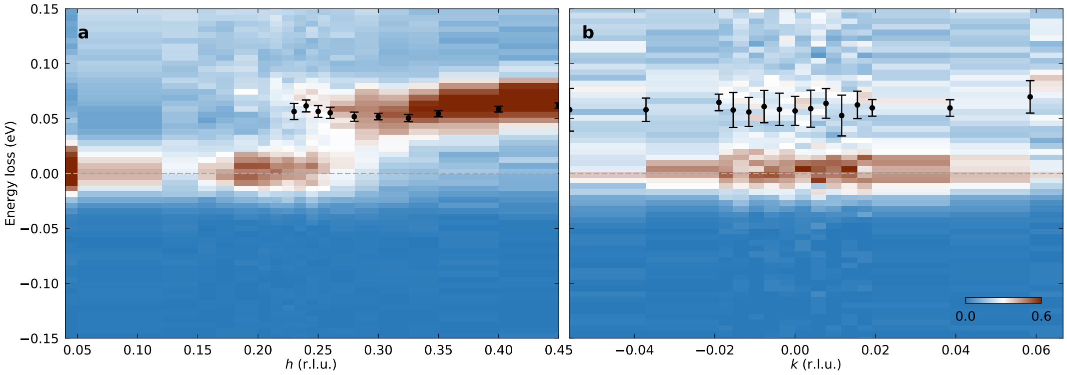

Supplementary Figure 3. **RIXS intensity recorded on LSCO x = 0.16**. **a** RIXS spectra probed in longitudinal h and **b** transverse k directions on LSCO $x = 0.16$. Data are recorded at a high-resolution beamline with a total energy resolution of 33 meV and at a temperature of 35 K. Horizontal dashed lines mark the zero energy loss. The black circles mark the phonon dispersion determined from fitting the spectra. Error bars are set by standard deviation from fitting.

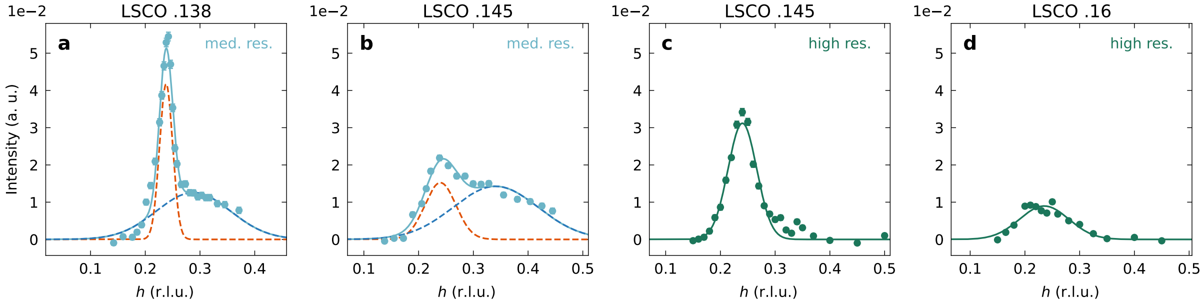

Supplementary Figure 4. **Charge order parameters**. **a-d** show the relevant elastic scans for the extraction of incommensurability δ, correlation length ξ (defined as the inverse half-width-half-maximum) and integrated diffracted intensity (amplitude I divided by ξ^2) shown in Fig. 4a-c of the main manuscript. For the medium resolution data, the scan contains a contribution from phonon scattering, therefore two components have been used for the fit. The red component shows the scattering from charge order, from which its parameters have been extracted. Error bars are set by counting statistics.

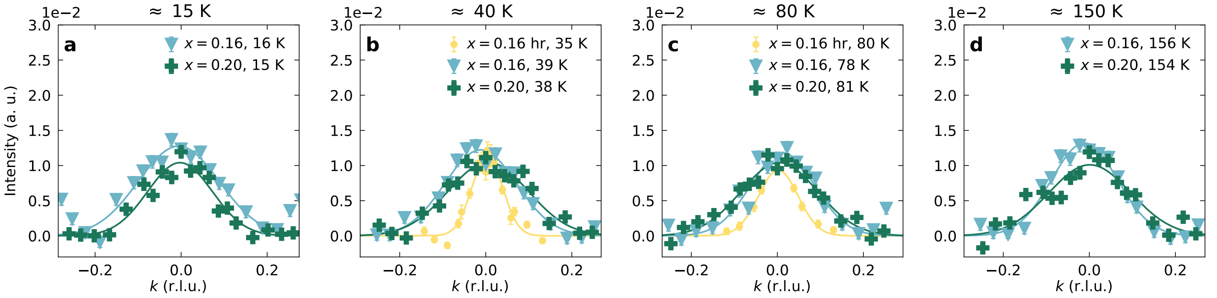

Supplementary Figure 5. **LSCO** $x = 0.16$ **and** 0.20 **temperature independence**. **a-d** compare the two overdoped LSCO $x = 0.16$ and 0.20 for different temperatures as indicated. High-resolution data on $x = 0.16$ is included in **b** and **c**, showing how the improved resolution results in a narrower peak while keeping the same amplitude. Error bars are set by counting statistics.
